# An online model composition tool for system biology models

**DOI:** 10.1186/1752-0509-7-88

**Published:** 2013-09-05

**Authors:** Sarp A Coskun, A Ercument Cicek, Nicola Lai, Ranjan K Dash, Z Meral Ozsoyoglu, Gultekin Ozsoyoglu

**Affiliations:** 1 Electrical Engineering and Computer Science Department, Case Western Reserve University, Cleveland, OH, USA; 2 Lane Center for Computational Biology, Carnegie Mellon University, Pittsburgh, PA, USA; 3 Department of Biomedical Engineering, Case Western Reserve University, Cleveland, OH, USA; 4 Department of Physiology, Medical College of Wisconsin, Milwaukee, WI, USA

**Keywords:** System biology models, Simulation, Composition, ODE solver

## Abstract

**Background:**

There are multiple representation formats for Systems Biology computational models, and the Systems Biology Markup Language (SBML) is one of the most widely used. SBML is used to capture, store, and distribute computational models by Systems Biology data sources (e.g., the BioModels Database) and researchers. Therefore, there is a need for all-in-one web-based solutions that support advance SBML functionalities such as uploading, editing, composing, visualizing, simulating, querying, and browsing computational models.

**Results:**

We present the design and implementation of the *Model Composition Tool (Interface)* within the PathCase-SB (PathCase Systems Biology) web portal. The tool helps users compose systems biology models to facilitate the complex process of merging systems biology models. We also present three tools that support the model composition tool, namely, (1) *Model Simulation Interface* that generates a visual plot of the simulation according to user’s input, (2) *iModel Tool* as a platform for users to upload their own models to compose, and (3) *SimCom Tool* that provides a side by side comparison of models being composed in the same pathway. Finally, we provide a web site that hosts BioModels Database models and a separate web site that hosts SBML Test Suite models.

**Conclusions:**

Model composition tool (and the other three tools) can be used with little or no knowledge of the SBML document structure. For this reason, students or anyone who wants to learn about systems biology will benefit from the described functionalities. SBML Test Suite models will be a nice starting point for beginners. And, for more advanced purposes, users will able to access and employ models of the BioModels Database as well.

## Background

Systems biology researchers have built, over the years, a large number of computational biological models, and, these models are recently becoming available in web-based data repositories such as the BioModels Database [1] and CellML Model Repository [2]. These web-based data repositories store hundreds of computational models, and provide manual curations of some of the models submitted by researchers.

Systems Biology Markup Language (SBML) is a standard for exchanging and storing biological-biochemical models. The majority of SBML models published to date involve specific and, most of the time, small biological sub-networks of organisms. Nonetheless, creating more complete or larger models of biological networks and simulating their behavior on a wider biological network provides a better understanding of how networks interact with each other. With the increasing number of models being published, there is a need to (1) compose larger models out of the existing models, and (2) simulate them on the spot, and on the web, if possible.

Randhawa, R et al. [3] proposed windows-based modeling tools to develop new models from the combination of multiple models. They define three different operators to combine SBML models, namely, *Fusion, Composition*, *Aggregation and Flattening*[4]*.* Partial implementations of these approaches can be found at JigCell’s web site [5].

Other groups proposed different composition tools such as SBMLmerge [6], sematicsSBML [7], and plugins for CellDesigner [8] for SBML Levels 1 and 2. The online version of semanticSBML, the successor of SBMLmerge, allows users to combine biochemical networks with identical species and/or reactions. Although the current version has limited functionality, it provides unique features to set and edit annotations of the models during the merging process. Furthermore, the plugins for CellDesigner provides a user friendly graphical interface for model composition. However, CellDesigner is not web-based.

More recently, SBML Level 3 core was released with new specifications to enable users to perform hierarchical composition and exchange of SBML models [9]. To compose and analyze SBML models defined with different SBML packages, there is a need for an integrated web-based environment and multiple tools, which provide:

•Advanced editing capabilities to redefine model elements,

•Simulation (ODE solvers) to quantify biochemical and physical processes,

•Visualization capabilities to identify metabolic network characteristics, and

•Selection of different computational models from a repository database.

Towards fulfilling the above-mentioned needs, we have developed an all-in-one web interface to compose new models from models defined in SBML format as a tool within PathCase-SB [10,11]. For the PathCase-SB Composition Interface, we use an approach similar to Fusion, and employ no additional non-standard SBML syntax in the combined model. In particular, the composed model contains all information of the submodels used in the composition process without any redundancies, although the information related to the relationship between elements of the composed model and the submodel is lost. When two models are combined, the simulation interface is available to simulate the composed model without any additional steps. As the composed model follows standard SBML specifications, visualization interface works on the composed model on the fly. The visualization tool uses yFiles library, which returns the layout to be drawn [12]. Details of the visualization interface have been described in Elliott et al. [13] and in Coskun et al. [11].

Since the Fusion approach is not reversible, our composition interface provides simulation, visualization, and SBML output of models being combined to help the modeler during the composition step. A semi-automatic matching algorithm (AutoMerge) for name overlapping in the models is used to combine the models. AutoMerge applies a MIRIAM annotation-based [14] and exact name matching-based algorithm to merge SBML elements and prevent SBML element duplication.

We have released two versions of the web site. The first one [15] hosts BioModels Database models, and the second one [16] hosts SBML Test Suite models. While the former one provides a system where advanced users can merge complex models published in the BioModels Database, the latter one provides basic models for beginners to practice and learn the composition process, or for any researcher who wants to understand the specifics of the composition algorithm.

The new features of the proposed Model Composition Tool are:

•*All-in-one hierarchical model composition capability and details of how it works.*

○ Describing how each process (merging and simulation, iteratively) is done, and

○ Giving specific examples at the very end that show how the model composition tool works.

•*Evaluation of the tool via models from both the BioModels Database (as uploaded into PathCase-SB database) and the SBML Test Suite.*

•*MIRIAM annotation-based and exact name-based species/reaction/compartment matching modules.*

•*Discussion of the architectural advantages of PathCase-SB as applied to the Model Composition Interface* in terms of s*upport for multiple simulation engine use*. The simulation engine (currently, RoadRunner) can easily be replaced by another simulation engine, so long as the new engine does provide a web service functionality.

•*Comparison* of our model composition approach with other systems, and discussing our design decisions.

•*Introduction of our clone web-site which only hosts SBML Test Suite models*. We believe that this site may have educational use, and may allow researchers to experiment with the model merging component.

## Implementation

In this section we describe the details of the model composition interface, the merge algorithm and the model simulation interface.

### Composition interface

**Figure 1:**
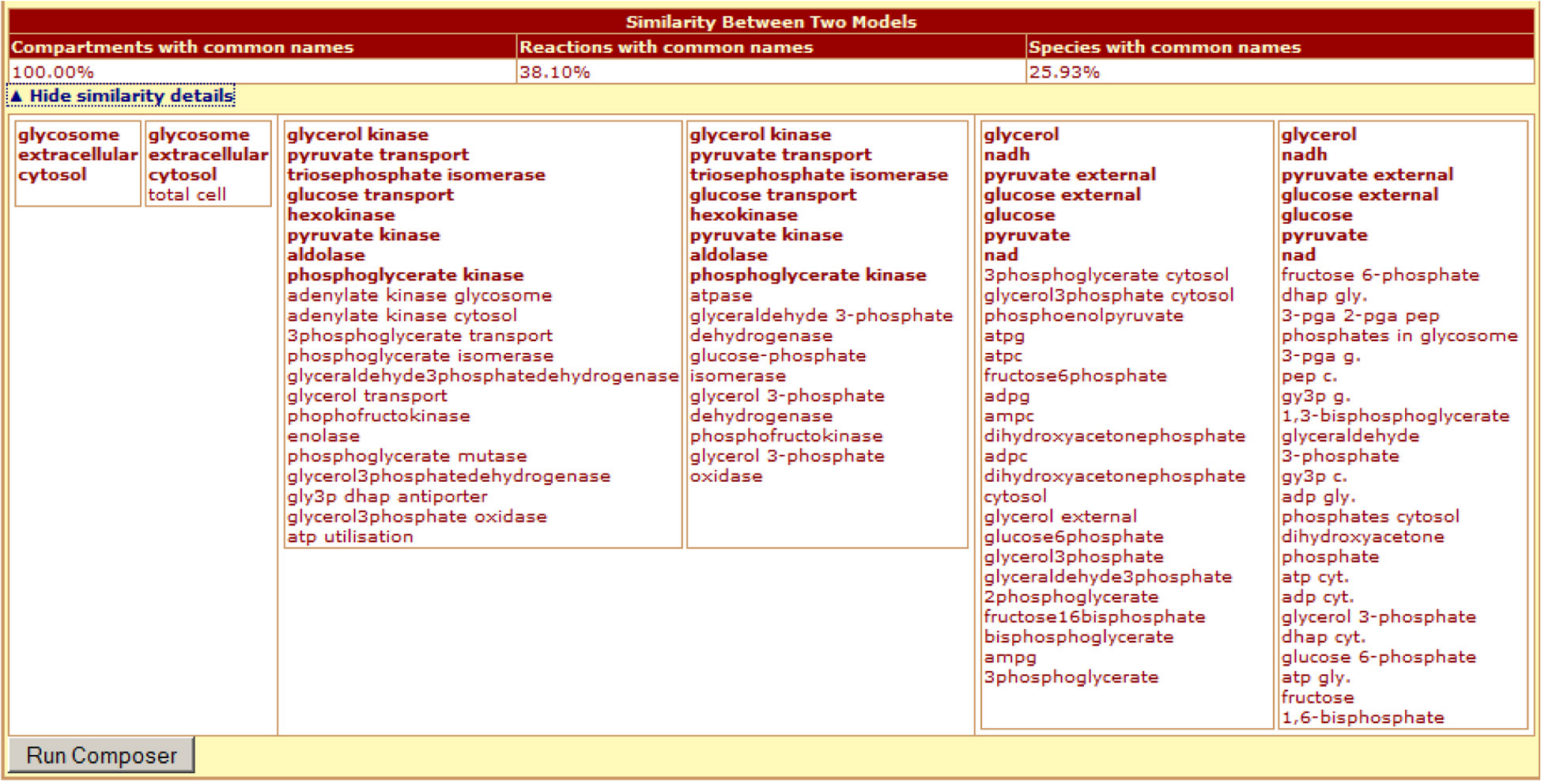
**Details of similarity between two chosen models.** The first column represents the Albert2005_Glycolysis model [10], and the second column represents Bakker2001_Glycolysis [11]. Compartment, reaction and metabolite name matches are displayed as percentages on top. Then, matching names are listed per entity.

In this subsection, to illustrate and explain the interface components, we apply the model composition tool to two sample models [17,18]. The PathCase-SB Model Composition Interface provides a three-step model composition process for computational models defined in SBML format. After clicking the “Compose Models” link on the main page, the user selects two models to be combined. The models can be uploaded by the user, or selected from models stored in the PathCase-SB database. As of January 2013, there are 366 parsed SBML models on PathCase-SB database; and testing each model from this relatively large dataset may be time-consuming to users. To help researchers with the model selection, PathCase-SB provides extensive browsing capabilities for parsed computational models via the PathCase-SB browser interface. In addition to this functionality, the model composition interface provides “similarity indices” between two pre-parsed models in order to aid researchers to pick the most appropriate model for their needs. Percentage similarities are presented based on exact matching of names/ids for Compartments, Reactions and Species elements in the models. Users can see the details of the similarity by clicking on “Show similarity details” link, which displays three consecutive sections, representing compartment, reaction and species as shown in Figure 1.

Before running the automated composition algorithm, there is an intermediate step in which users can manually match compartments, species and reactions. Details of the chosen models are displayed underneath the matching table as a reference. One SBML element (compartment, species and reaction) cannot be matched with multiple elements. One-to-one matching is enforced by the user interface because the same SBML element with different names should not exist in an SBML model. If the user does not want to do manual matching, all dropdown lists need to be set to the “Automatic” option. In the “Automatic” option, the composition tool automatically matches compartments, species, and reactions according to their original naming conventions.

Users then employ the tools of the interface, and edit the merged model in a “tree list view” or “textbox view”, and update the resulting model. After running the composition tool via the “Run Composer” button, *AutoMerge* algorithm (to be described in *The Merge Algorithm* subsection) is executed.

**Figure 2:**
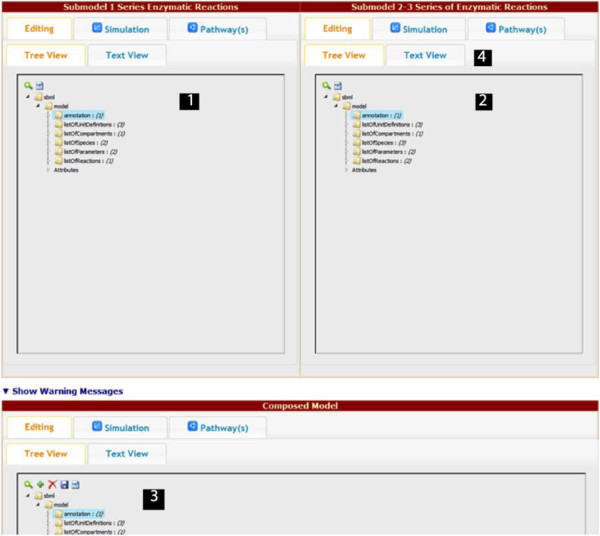
**Overview of model composition layout and tab views. 1** and **2** show the hierarchy of each corresponding model to be composed. **3** shows the panel for the composed model. **4** shows available tabs for the second model.

In the last step, the user can see the complete set of available composition elements in a single page, as shown in Figure 2. In this step, the user can (a) edit the auto-merged SBML file, (b) alter the numerical values assigned for species, boundary species, and parameters in model simulations, (c) study the visualization graphs, and (d) observe the warning messages (and take actions, if needed). The user can examine the models (arrows 1 and 2 in Figure 2) being combined, and the composed model (arrow 3 in Figure 2) separately. Each of these three model user interfaces are independent from each other, and all provide support for the four components, namely, the Editing tab which consists of the Tree View (searchable a hierarchical XML representation of the SBML model) and the Text View (text editor for raw XML text of the original SBML file), the Simulation tab (simulation results for the model), and the Visualization tab (applet-based visualizations of the two models). All tabs are provided for the composed model as well, after the merging is complete.

### Technical details

Tree view at PathCase-SB *Composition Interface* is powered by an open source XML Library [19] and the code is modified for SBML composition. On the client side, jQuery, jsTree and jQuery UI libraries are employed to display a hierarchical view of SBML models. Client controls invoke ASP.NET Web Methods and Web Services at the backend via AJAX programming. Text view of the PathCase-SB *Composition Interface* is a basic server side TextBox, which synchronizes with tree view modifications on the client side.

PathCase-SB *Visualization Interface* is powered by PathCase-SB *Graph Viewer* (a client-side java applet) that produces interactive pathway graphs, biochemical network graphs modeled by systems biology models, or both, with various mappings between them. The visualized model network and/or pathway can be rearranged manually or automatically, zoomed in/out, panned, expanded/collapsed, queried from, saved locally as jpeg file, and studied in detail.

### Merge algorithm

The *AutoMerge* algorithm attempts to perform an exact match on compartments, species, and reactions of the two models being composed. Matching process works with (1) MIRIAM annotations of the SBML elements (whenever available), and (2) the *name* attribute of the SBML element. Below we discuss the matching algorithm of these three SBML elements in detail.

Note that, in order to properly perform model composition, properties such as parameters, kinetic laws, events have to be created. The system has special rules for the creation of these properties.

To compare SBML models element by element correctly, models should be *compatible* in terms of their SBML Levels. Otherwise, the structure and attributes of specific XML elements may differ in different levels [20]. *AutoMerge* assumes that SBML models being composed are *compatible* and created following correct SBML guidelines.

AutoMerge checks for matches in species, compartment attributes, *sboTerm* attributes (if available) for species, and reactions. Details of the matching are discussed next.

*Compartments*. A standard SBML element for a compartment contains *id*, *name* and, optionally, *sboTerm* XML attributes that are used for comparison. Compartment matching is based on the *annotation* and *name* attributes is accomplished as follows:

1. If the *name* attribute of compartment elements match or there is a shared MIRIAM annotation among two compartment elements, merge the compartments into one compartment via pivoting the first model. XML elements such as *name*, *units*, *size* and *sboTerm* of the first model override the XML attributes of the second model, and these overwrites are displayed in the warnings section if the values are different.

2. Otherwise, do not merge the compartments, and add both compartments as separate compartments into the composed model definition.

*Reactions*. For reaction elements, attribute information about *annotation*, *name*, *reactants* and *products* for each reaction of the first SBML model are compared and matched to those of the reactions in the second SBML model, in order to identify whether the two SBML models share common reactions. While comparing two reactions, *AutoMerge* first checks *name* element similarities, and then reactants and products of the reaction. Reaction matching is accomplished as follows.

1. Merge the two reactions (say, R1 and R2) into one when the following three conditions are satisfied.

a. R1 and R2 have a shared MIRIAM annotation,

a. The *name* elements of the reaction R1 and R2 are the same,

a. The *list of reactant(s)* of the reaction R1 and R2 are the same,

a. The *list of product(s)* of the reaction R1 and R2 are the same;

2. Otherwise, do not merge reactions, and generate two different reactions in the composed model definition.

The composition prepends the model identifier as a prefix to the reaction name of the second model. Therefore, even when the *id*s of the reactions are the same, they do not override each other. For instance, if reaction R1 exists as a name in both models and they do not match, *AutoMerge* does not change reaction *name* and *id* attributes of the first model, but changes the reaction *name* and *id* attributes of the second model to *id-of-model2_*R1, given *id-of-model2* is the identifier of model 2.

In order to apply the matching rules, *AutoMerge* first parses the *name* attribute of reaction, and then children XML elements of listOfReactants and listOfProducts tags to find reactants and attributes, respectively.

*Species*. Matching is based on *name* attribute, and MIRIAM annotations. If the same species are named with the same convention or have a shared annotation, automated algorithms can easily match these two species, merge them, and the merged species entity into the composed model.

For species, *AutoMerge* checks two XML attributes while comparing them, namely, *name* and *compartment*. Species matching is accomplished as follows.

1. If both *name* and *compartment* attributes of two species match, or two species have a shared annotation, merge the two species into one. Value of the initial concentration attribute of the first model overrides the second model; but, if values are different in the two models then this merge information is displayed in the warnings panel.

2. Otherwise, insert both species as different species, together with their compartment information, into the composed model definition.

In order to differentiate references to different libraries, the XML specification uses *namespaces* defined by *xmlns:m* attribute. While combining two models, *AutoMerge* creates a union of available *namespaces* in the models being compared.

As *AutoMerge* applies the above-listed rules, some parts of SBML documents may not merge correctly. In that case, the model composition interface provides a list of *Warning Messages*. During comparison, some XML attributes such as *unit*s, *initialAmount*s, and *stoichiometry* may not match even when the *id*s and *name*s of XML elements do match. When the model composition interface merges elements with differing attribute values, a list of warnings is displayed. In such cases when two merged elements have different values for the same attribute, the value in the first model is used for the composed model.

In addition to attribute value conflicts, *name* conflicts are displayed as warning messages as well. Assume two reactions whose *id*s are both *reaction2*; and they are different reactions. *reaction2* elements will not be merged in the composed model, but their original *id*s cannot be kept in the composed model either since SBML defines *id* attribute as a unique identifier for each model. For this reason, “id-of-model2_” prefix is added to the *id* in the second model, which refers to *reaction2* of model 2.

### Simulation interface

The interface (1) uses SBML files as direct input to the simulation process, since the models are exchanged and made available in SBML, and (2) integrates an existing stand-alone simulation engine, namely, RoadRunner [21], which is one of the sophisticated and highly-capable simulation engines available to the research community.

There are many simulation tools for SBML documents as covered in detail in SBML Software Matrix which compares SBML software [22]. Among those, some of the software is not free; some of them are working only on Windows operating system; and some of them do not provide all the capabilities needed by our interfaces. For client-based solutions, installing the correct updates of the client software is both a time-consuming and difficult task. Therefore, for easy distribution and updates, we have chosen to build a web-based solution. The web interface is updated on the server side. Moreover, users can access the tools from everywhere with no machine/OS compatibility issues. As of January 2013, there exists a few web-based simulation applications; but simulation editing capabilities in Reactome [23] are very limited, CytoSolve [24] cannot simulate some BioModels verified models, and maintenance of JWS Online [25] is stopped after 2007.

For PathCase-SB, the web based simulation interface is built and integrated to the PathCase Systems Biology web site. In the simulation interface, users can re-simulate the computational model via:

1. Changing numerical values of parameters,

2. Selecting and modifying initial concentrations or amounts of species and boundary species,

3. Choosing metabolic fluxes to plot,

4. Changing start and end values of time period (time scale is specified by the model),

5. Modifying tolerance values for absolute tolerance and/or relative tolerance,

6. Changing the number of data points to plot on to the graph (proportional to specified time period),

7. Adding user generated experimental value sets, and

8. Observing the results of the new simulation on the fly.

Experimental results are manually editable on the textbox field specified for users during the tests. Users can find model details (such as the version of the model, notes by the author, and defined units) above the simulation graph.

From the simulation interface, the user can define custom numerical values for basic simulation settings, which reside underneath the plotted graph in three columns. Start and end values of the time period specify the interval that is plotted onto the simulation graph. These two integer values are in the unit of time scale specified in the model.

The “number of data points” field defines the frequency of data points in the plot of the simulation graph. The more the data points there are, the smoother will be the simulation plot.

The rightmost column in the basic settings contains absolute and relative tolerance values for the simulation engine. By default, RoadRunner sets these values to 10^-16^ and 10^-6^ for absolute and relative tolerances, respectively. For these two very small floating number values, scientific notation needs to be used to save space. These values are specified in E notation (e.g. 1E-16, 1E-6) and saved globally during user’s session.

**Figure 3:**
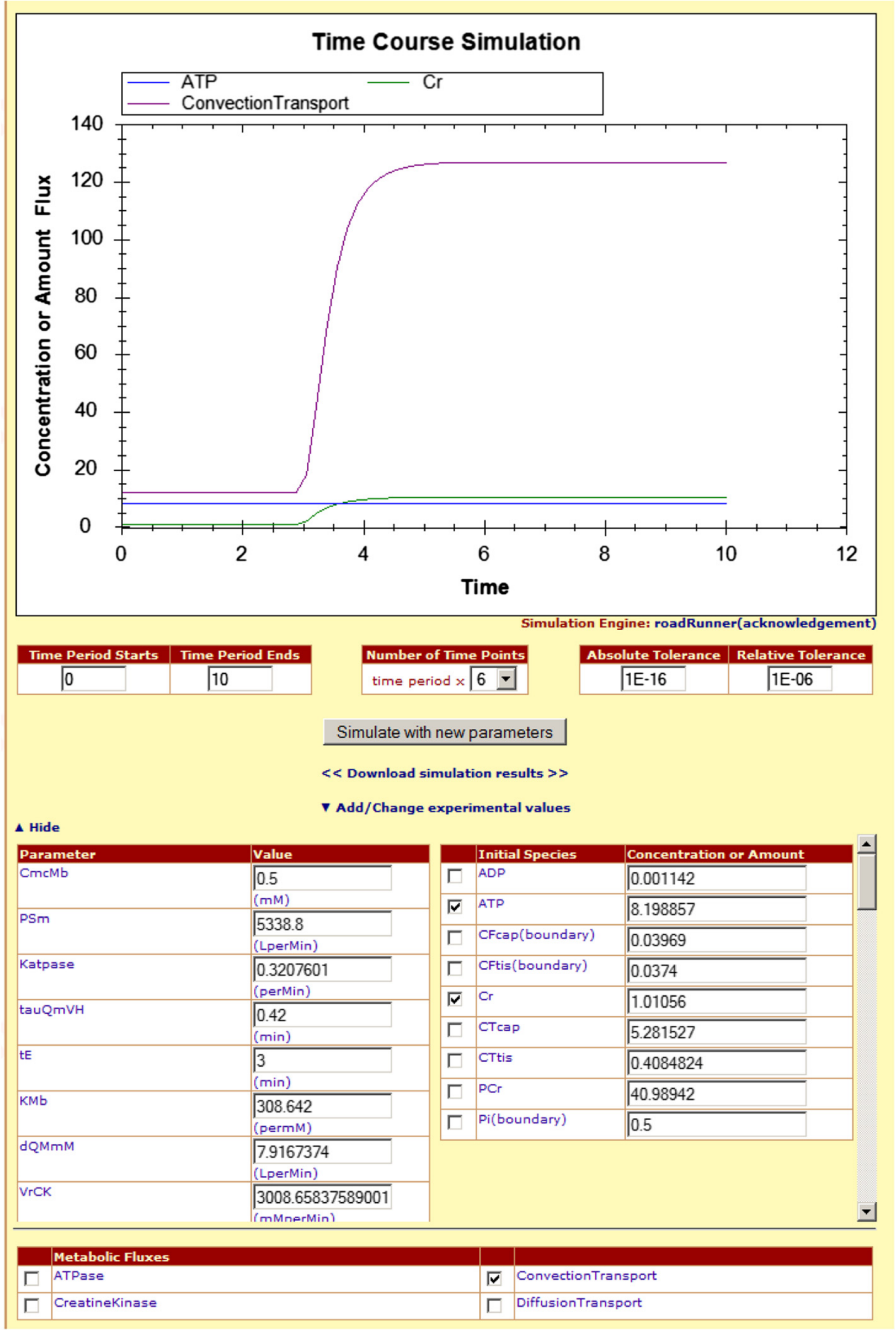
**An example of a simulation result is shown.** Species and boundary species are listed with their initial concentrations or amounts. They can be edited by the user in order to perform a new simulation. Only the species and/or boundary species whose checkboxes checked (in this case only two species and one flux) are selected.

As illustrated in Figure 3, once the user expands “Change parameters and initial concentration or amount of species” link, a list of options for available species, boundary species, parameters, and metabolic fluxes appears. User can modify the parameter values from the left column of parameters table, and also access the unit for each value (shown next to each field in parenthesis). Once the value of the parameter is changed from the value specified in the model file, the original value can be seen by going over to the specific parameter’s value field with mouse pointer.

On the right column of the panel in Figure 3, species and boundary species are listed with their initial concentrations or amounts. Only those species and/or boundary species for which checkboxes are checked are plotted onto the simulation graph. At the start of the simulation interface, all species are unchecked; and, in the case of Figure 3, all of them are plotted to the graph. The initial concentrations or amounts can be changed to positive rational numbers only.

In addition to species, boundary species and parameters, users can also plot metabolic fluxes onto the simulation graph. The metabolic flux list resides under the parameters list, and each of these metabolic fluxes has a checkbox next to it as shown in Figure 3. Users can select and deselect to show or hide the metabolic fluxes on the simulation graph.

**Figure 4:**
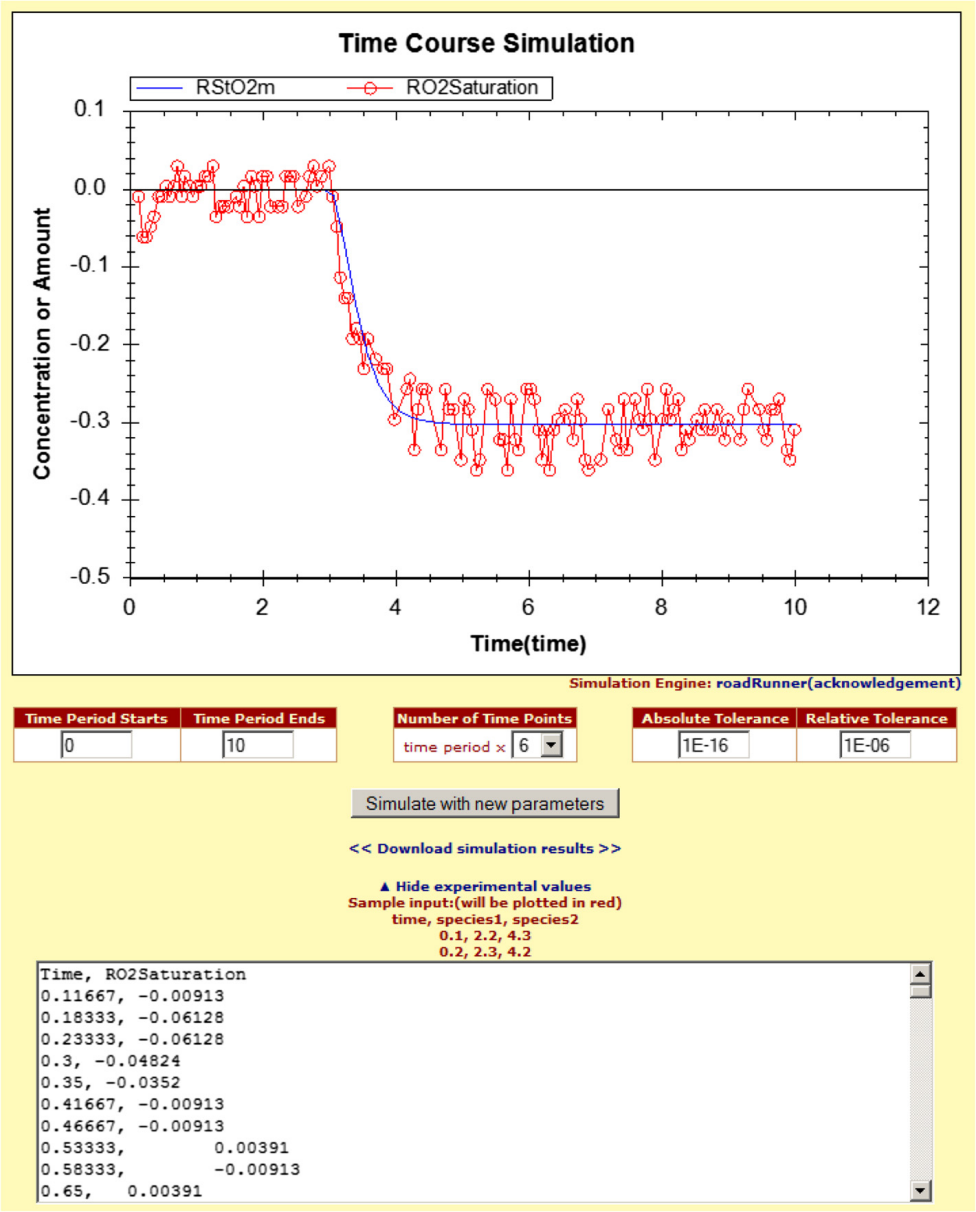
Simulation plot compares the concentration change in the species RStO2m calculated according to the model with the input experimental RO2 Saturation data to validate the model.

Modelers may also want to compare their data, possibly prepared after conducting lab experiments, with a currently curated and verified model. This way, overlapping data points between experimental data and the original model simulation data can be compared easily. In the simulation interface, users can enter experimental values into a large text field by clicking the “Add/Change Experimental Values” link as illustrated in Figure 4.

Since the majority of applications (MATLAB, Excel, etc.) that researchers use during their experiments can export data into a comma-separated value (CSV) file, our simulation interface accepts valid CSV file content as input. The data, which is specified in the text field, needs to be a set of time course values. The first value in the comma-separated list has to be time, and the following values can be the amount of species, boundary species, or metabolic fluxes in the experimental data. These values can be modified manually, and simulation can be rerun by clicking the “Simulate with new parameters” button. To differentiate experimental data from the original plot, only the experimental data is plotted in red color in the simulation graph by default.

Implementation of the simulation interface is programmed with Microsoft.NET Framework using the C# and ASP.NET languages. User interface itself uses the asynchronous JavaScript and XML (AJAX) technology in order to achieve better performance and seamless user experience on simulation interface. Simulation uses localized (running locally) RoadRunner application programming interface (API) on the backend. A third party library called ZedGraph [26] is used to plot simulation results onto the graph.

The modular implementation (built as a user control) of the simulation interface provides easy integration to other parts of the PathCase Systems Biology web site such as SimCom and iModel tools.

### SimCom--simulate and compare computational models side by side

PathCase Database currently hosts KEGG pathways along with BioModels models. Two data sources are integrated as described in [10]. We use own parser to map the KEGG pathways in KGML format to our data model [13]. There are two other major convertor tools such as KEGGconvertor [27] (a java based tool to convert KGML to SBML) and KEGGtranslator [28] (a java based tool to convert KGML to various formats such as BioPAX, SBML and GML). However, we have elected to implement our own parser to populate our own database schema, which is designed for integrating different data sources in an extensible and flexible way.

For some KEGG pathways, there are multiple BioModels models. Therefore, side by side comparison of these biological models (for a single pathway) can allow researchers to identify the main similarities/differences between such computational models. The SimCom tool provides the functionality to simulate up to four models for the same pathway side by side (in new pop-up windows) from the PathCase-SB web site.

Once the user selects a pathway from the dropdown list in the SimCom tool, a model list for the selected pathway is loaded automatically. In square brackets, organism information for each model, if available, is displayed next to each model. After selecting models to compare, user can simulate all selected models side by side by clicking on the “Simulate Selected Models” button. For each selected model, an “independent” fully functional simulation interface is loaded. Since the simulations are independent from each other, the user can close one without changing the state of the other simulations, and continue modifications on the currently open simulation interface popup windows.

### iModel--simulate user uploaded computational models

Currently, when a researcher receives/downloads SBML files, (s)he needs to download software to analyze the models on a machine. For a quick editing or a quick look at simulation results and/or visualization of the computational model, different types of software need to be installed onto client computers. After installing and setting up the OS-compatible software, users can upload their model and see the simulation results or other functionalities. There are two major bottlenecks: (a) finding the correct/compatible client software is not always an easy task; and, (b) keeping the client software up-to-date is another challenging task.

The iModel tool allows users to upload their own SBML models into the PathCase-SB site to simulate and visualize their models. First, uploaded models are parsed with the PathCase SBML Parser, which uses the libSBML library [29] in the backend. After being parsed, uploaded models are stored in a separate temporary database. Therefore, uploaded models are not maintained or kept in the PathCase-SB database for privacy issues. Currently, iModel accepts only XML file types of up to 5 MB in size to upload. If the uploaded model has syntax errors, or in an incorrect format, or, for any reason, it cannot be parsed correctly, iModel will indicate to the user that the model is incorrect by an error message.

By using the “Choose File” button in iModel, users can browse their local hard drives, and choose the SBML model file to be uploaded. Users can then click on the “Upload My Model” button to initiate the parsing process. If the model is uploaded and parsed successfully, users can visualize and simulate the model.

## Results

In this section, we discuss empirical evaluations of the Simulation Interface and the Composition Interface separately with different test model inputs. Hardware configuration of Intel Xeon 2.27 GHz (2 CPU) installed with 6GB RAM PC is used during the experiments of this chapter. All experiments are conducted on a 64-bit Windows 7 OS with Mozilla Firefox 20.

### Simulation interface experimental setup

To test the simulation interface, we have used models from the literature [1], each having different levels of complexity in describing metabolic reactions and transport processes in physiological systems. Each physiological system is described by a set of Ordinary Differential Equations (ODE) that represents the mathematical model. In order to compare simulation results of kinetic processes of the system, the mathematical models are solved using RoadRunner [21], COPASI [30], JSim [31], iBioSim [25], and Jarnac [32] simulation engines.

For each test case, simulations of the computational models are obtained using the above-mentioned simulation environments with specific versions as shown in Table 1. Different absolute tolerance and relative tolerance values (10^-3^ - 10^-8^) are used to assure that the solution has converged to that obtained with MATLAB. The SBML Test Suite Database [33] could be also used as valuable tool to compare simulations obtained with different software systems beside those listed above. It should be noted that the evaluation of different software systems is limited to the models available in the database.

**Table 1:** List of tested computational model simulators with version information

**Simulator**	**MATLAB**	**COPASI**	**iBioSim**	**JSim**	**RoadRunner**
**Version**	7.10	4.5.30	1.3	1.6.94	2.8.1

### Composition interface experimental setup

In order to evaluate the model composition interface and the rules as defined in Chapter 4, there was a need to use multiple models with minor differences. For this purpose, we have used the online model repository of 951 SBML models (compatible to our parser), which were used to test simulation engines at the SBML Test Suite [33]. This model set contains different combinations of few reactions and a few species with different kinetic laws, events, parameter values and so forth. The composed models are not very large in size, and, therefore, the composition results can be and have been validated easily by biochemistry experts in our research group.

### Simulation interface experimental results

In this section, we present the test results of the simulation interface with two models by Hucka et al. [9], and Vicini and Kushmerick [34].

### Example 7.1 In Hucka et al. - Kinetics of unireactant enzyme

In this example, the rate process for a unireactant enzymatic reaction is presented. ES (enzyme substrate complex) is formed from the reaction between E (enzyme) and S (substrate). Following reaction breaks down ES to form E (free enzyme) and P (product). This enzymatic reaction formula is represented as *E* + *S* ⇆ *ES* → *E* + *P*.

In order to represent reversible reaction in SBML document, *reversible* attribute in *reaction* tags is used.

**Figure 5:**
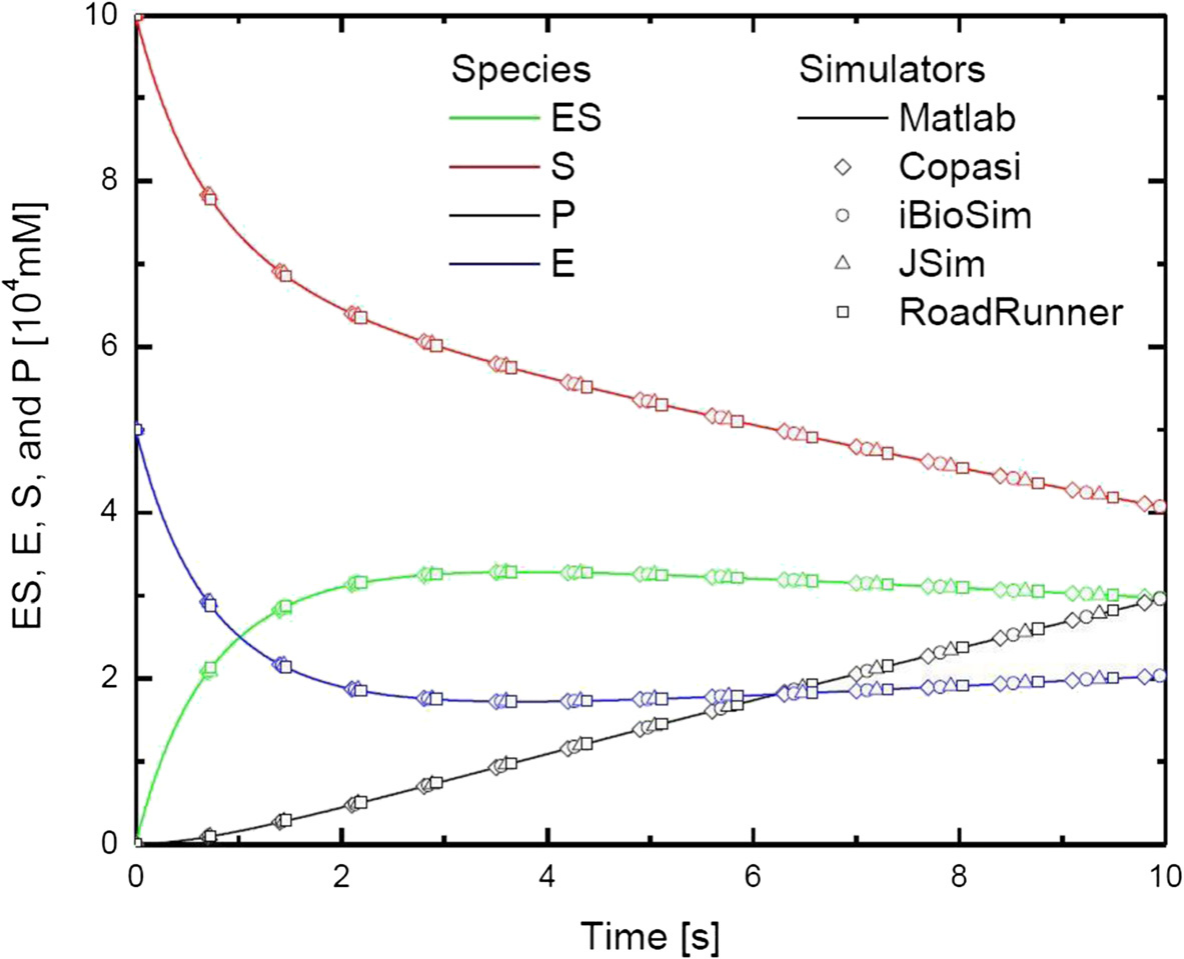
**Simulation results for species E, ES, S, and P in **[20]** using 5 different simulators.**

Simulation results from different simulation engines for species ES, S, P and E are displayed in Figure 5. Unique data point shape is printed for each simulator on the graph, and the simulation results overlap with each other for all the simulation engines. We have applied different numerical values for relative tolerance and absolute tolerance between 10^-3^ and 10^-6^ during our tests. Regardless of the tolerance values, RoadRunner SBML model simulation results used by PathCase-SB simulation interface are consistent with the other simulators.

### Example in Vicini and Kushmerick - Cellular bioenergetics

In this example, we use the model by Vicini et al. [34] which measures muscle ATP utilization and synthesis rates during muscle stimulation in human body. These enzymatic reaction formulas are represented as: *O*_2_ + *ADP* + *P*_*i*_ → *ATP* + *H*_2_*O* , *ATP* → *ADP* + *P*_*i*_ , and *ATP* + *Cr* ⇆ *PCr* + *ADP* + *P*_*i*_. These reactions (oxidative phosphorylation, ATPase, and creatine kinase respectively) regulate the ATP homeostasis during muscle stimulation.

When ATP breaks up into ADP and an inorganic phosphate, cellular energy is released. CKase is used to keep the equilibrium between ATP and ADP when high amount of cellular energy is required. With the help of O_2_, oxidative phosphorylation generates ATP as the primary energy source process.

**Figure 6:**
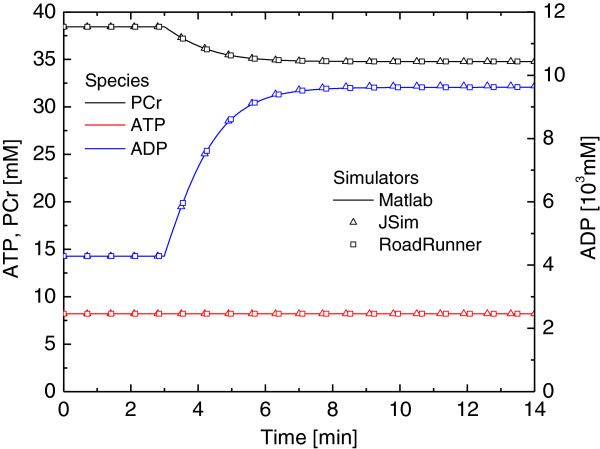
**Simulation results for species PCr, ATP and ADP in **[34]** using 3 different simulators.**

In Figure 6, simulation results of PCr, ATP, and ADP from MATLAB, JSim and Roadrunner simulation engines are displayed. In order to compare the results, we have modified the relative and absolute tolerances of simulators as follows: MATLAB (10^-6^), JSim(10^-3^) and RoadRunner (10^-5^). MATLAB can provide similar results for relative and absolute tolerance values in the range of 10^-1^ -10^-6^, nonetheless, RoadRunner has produced computational model simulations comparable with MATLAB results for the tolerance values between 10^-4^ and 10^-5^. The simulation results provided by RoadRunner are very similar to the ones produced by MATLAB for the species in both Figure 5 and Figure 6. Absolute tolerance is set to 10^-8^, and relative tolerance is set to 10^-2^ to produce these figures. In our tests, accuracy of the model simulations obtained with PathCase^SB^ simulation interface is equal to other SBML simulators.

### Composition interface experimental results

We have used SBML Test Suite to test the composition interface. SBML Test Suite is a platform for developers to test their simulation tools, providing around 1200 basic models and expected simulation results for verification purposes. In this database we identified 40 different groups of models according to the specific reaction as reported in Table 2. We have randomly picked pairs from Table 2 and verified the syntactic correctness (i.e., no manual checking is performed for semantic meaning) of the composed model. Below, we present the results for a randomly chosen model pair: model 15 (Group 7) and model 20 (Group 11) and then give statistical data about the composition interface.

**Table 2:** The model groups of the SBML test suite database classified by reaction properties

1. S1 → S2	21. S1+S2 → S3+S4, 2S3+S4 → S1+S2
2. S1 → S2, S2 → S1	**22.** S1 → S2, S2 → S3, S3 → S1
3. S1 → 2S2	**23.** S1+S2 → 2S3, S3 → S1+S2
4. S1 → 2S2, 2S2 → S1	**24.** X0 → T, T → X1
5. S1+S2 → S3, S3 → S1+S2	**25.** X0 → 2 T, T → X1
6. 2S1+S2 → S3, S3 → 2S1+S2	**26.** 2X0 → T, T → X1
7. S1+S2 → S3+S4, S3+S4 → S1+S2	**27.** S1+S2 → S3, 2S3 → S1+S2
8. S1+S2 → S3+2S4, S3+S4 → S1+S2	**28.** 2S1 → S3, S3 → S1
9. S1→S2, S2→S1, S2→S3+S4, S3+S4→S2	**29.** S1+S2 → S3, S3 → S1+2S2
10. S1+S2 → S3, S3 → S1+S2, S3 → S1+S4	**30.** S1+S2+S3 → S4
11. S1 → S2, S2 → S3, S3 → S4	**31.** S1 ↔ S2
12. S1 → 0.3S2, 0.7S2 → S1	**32.** S1+S2 ↔ S3
13. S1+S2 → 2S2, S2 → S3	**33.** S1 ↔ S2+S3
14. S1+S2 → 2S2, S2 → S3, S3+S4 → 2S4	**34.** S1 ↔ 2S2
15. S1 → S3, S3 → S1	**35.** S1+2S2 ↔ S3
16. S1 → S3, S3 → S2	**36.** S1 ↔ S2+2S3
17. S1+S2 → S3, S3 → 2S1+S2	**37.** S1 ↔S2, S3 ↔ S4
18. 2S1 → S2	**38.** A4 ↔ A2, A1+A2 ↔ A3
19. S1+2S2 → S3, S3 → S1+S2	**39.** A4 → A2, A1+A2 → A3
20. S1+S2 → 2S3+S4, 2S3+S4 → S1+S2	**40.** S1 → S2, 2S2 → S3, S3 → S4

### Composing model 15 and model 20 in SBML Test Suite

**Figure 7:**
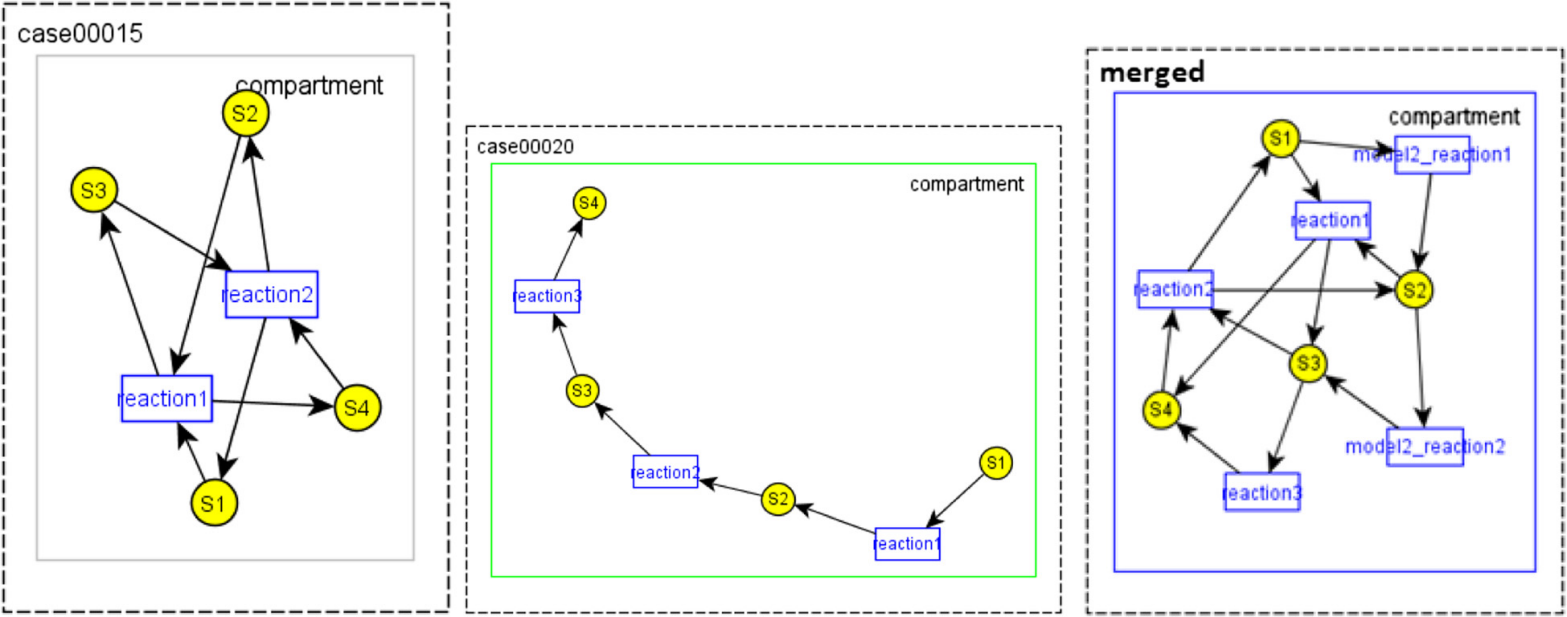
Visualizations of Case 15 (left), Case 20 (middle) and the composed model (right).

**Figure 8:**
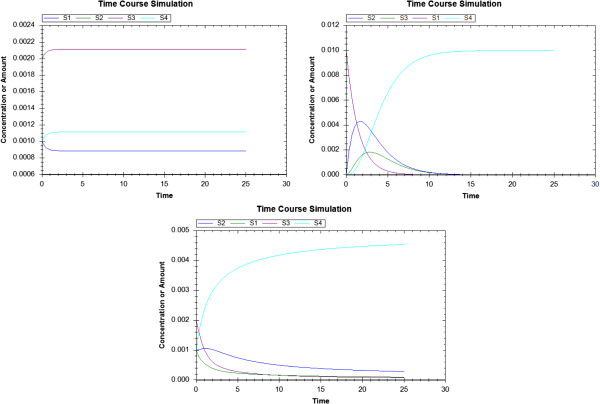
Simulation results of Case 15 (upper left), Case 20 (upper right) and composed model out od Case 15 and Case 20 (bottom).

When *AutoMerge* algorithm runs, none of the reactions merge although they have the same name because the substrates and products are different. Therefore, in the resulting model, we have a reaction named *case00020_reaction1* and *case00020_reaction2* in addition to reaction1 and reaction2 from Case 15. As the species S1, S2, S3 and S4 have the same ids, they are merged in the resulting model. Parameters are not merged although the *id*s are same, and resulting model contains *case00020*_*k1* and *case00020*_*k2* as the parameters for *case00020*_*reaction1* and *case00020*_*reaction2*. Visualizations of the models are shown in Figure 7, and simulations results are shown in Figure 8.

### Composition interface applicability statistics

While retrieving the performance statistics, we have tested the success rate of *AutoMerge* algorithm on randomly chosen 1,000 pairs of models from different reaction groups. These tests are also conducted without the user interface, and all run from the test console application. Overall, the automatic merge successful execution rate (syntactically) is 98.4%, and the remaining 1.6% needs manual corrections on the files, which are being composed. Thus, our conclusion is that *AutoMerge* works well with a very high success rate. However, we have not conducted a fully manual check for the correctness of the composed model as checking the correctness of a composed model semantically is a manual process.

## Discussion

### Model composition

In this paper, we have proposed a web-based tool to provide an integrated environment to edit, visualize, select computational models from repository databases such as BioModels and SBML Test Suite, compose new models and simulate them. Then, we have provided composition and simulation interfaces for SBML models. Our web-based tool can be iteratively used for both (1) models used for composition, and (2) the composed model in the same window to facilitate the work of the user during the model composition process. As previously highlighted in other works [6,7], there is a need for efficient algorithms with user-friendly interfaces during and after the model composition (merging) process. The reason is that users need to specify desired features of the composed model, and then resolve iteratively conflicting annotations. Our integrated web-based tool presents unique features to facilitate the model composition by an iterative process that also provides visualization of metabolic pathways and simulations.

As of July 2013, the current version of the Pathcase-SB visualization tool allows editing the model only after the composition process. Another group proposed [35] an alternative web-based solution, The BioGrapher, to provide metabolic network layout, editing and visualization tool, which supports Systems Biology Graphical Notation (SBGN). However, this tools is not currently integrated into a model composition tool. Model composition tools, such as semanticsSBML [7], has been released as an advancement over their predecessor SBMLmerge [6] to handle editing, verifying MIRIAM annotations and SBO terms in SBML models. Although it is a free software package that provides features for building, annotating, checking, and merging models, the web-based version has limited functionality.

Gennari et al. have proposed [36] a *SemSim* architecture that supports not only annotations, but also semantic information of the model that could be used for composition especially dealing with multi-scale and multi-domain modeling. Currently, the software *SemGen* allows automating annotation, composition and decomposition of *SemSim* model [37-39]. Although, this approach is quite promising, it requires the conversion of a model from SBML or CellML format into the *SemSim* format. Our main contribution is to provide an integrated web-based tool to deal not only with model composition, but also with visualization and simulation tools.

### Model simulation

Systems Biology Workbench (SBW) [21] provides an environment where different software tools can interact with each other over a common communication interface. SBW also provides wrapper classes for different programming environments, and thus can easily be ported into PathCase environment with minimal coding requirements. Therefore, we initially concentrated on using simulation tools that are compatible with the SBW environment. Among several alternatives, we have found that RoadRunner has the most robust event handling implementation. Hence, within SBW framework, we worked with RoadRunner.

As another alternative simulation environment, we worked with MathSBML [40] in the Mathematica environment. MathSBML is an open-source package that houses a library of functions to parse, create, simulate, and edit SBML model files within Mathematica. MathSBML also fully supports events.

CellDesigner [8] is a software tool which is compliant with SBW [16], and provides both extensive model editing and simulation features through third part tools such as Jarnac [32], MathSBML [40] and COPASI [30]. It also has a visualization interface, so that we can visually inspect the created models for correctness at the structural level. Through MathSBML, CellDesigner supports events. In the end, we decided on RoadRunner, which provides up-to-date.NET compatible wrapper classes in its API for remote procedure calls.

The composition of models from the BioModels Database is currently limited to the conflicting annotations used by users in developing their SBML models. In order to experiment with the composition tool we provide a separate site, which only hosts SBML Test Suite models. As they are clearly defined, and easier to understand, our clone site is a nice source to get used to the dynamics of the merging process and the algorithm. We think this feature is a nice contribution for the teaching purposes as well.

### Future work

We plan the following extensions to the Model Composition Tool.

*Approximate and score-based name matching techniques (via web services).* The capability of matching (i) species, (ii) reactions, and (iii) compartments is essential during model comparisons prior to model composition. Currently supported exact name matching and MIRIAM annotation-based matching modules are useful, but have limitations. Exact name matching does not perform well, even though it is useful on SBML Test Suite models, as naming conventions differ among the authors of BioModels models. And, currently, only 16% of models in PathCase-SB database (which are originally from the BioModels Database) have MIRIAM annotations. Therefore, approximate name-matching techniques from computer science, specialized for life sciences, is a desirable alternative. Towards that end, we have developed general-purpose basic bio-entity matching techniques. We are going to complete a web service functionality for approximate name matching of species, reactions, and compartments, to be used within the model composition tool as an additional matching tool.

*Enhancing the functionality of the model composition tool using the extensible architecture of PathCase-SB.* We list four extensions.

•*Support for multiple simulation engine use (via web services)*. The simulation engine (currently, RoadRunner) can easily be replaced by another simulation engine, so long as the new engine does provide a web service functionality.

•*Support for larger network comparisons for larger models via web services.* We are working on a web service functionality that compares networks or sub-networks (of models).

•*Support for CellML parsing via web services*. PathCase-SB is designed to support model composition for models designed in other formats, namely, CellML.

*Improving the AutoMerge algorithm* by optimizing various decisions it makes, such as, instead of giving the first model the priority, picking the entity with more information (e.g., annotation), and enabling the merge of units and parameters.

## Conclusions

PathCase systems are widely used by researchers. We expect that, after the addition of new capabilities, namely, (a) Model Composition Interface and (b) Model Simulation Tools into PathCase-SB, the usefulness and the user base of PathCase-SB system will increase.

These new tools and interfaces can be used with little or no knowledge of the SBML document structure. For this reason, students or anyone who wants to learn about systems biology will benefit from the functionalities such as model simulation, model composition, and pathways visualization of mathematical models.

Since the whole PathCase-SB web system is integrated, in the future, there is a need to test these tools and interfaces after any significant system or code updates in the future. For this reason, automated integration tests are developed in C#.NET via Selenium HQ software tool [41]. These tests are run via NUnit software [42], which checks multiple points of the web site, and verifies specific values on the web page.

As was discussed, the *AutoMerge* algorithm takes care of many issues that arise during model composition and simulation, which the modeler does not need to deal with. Nonetheless, for many complex composition tasks, cases such as (1) inconsistent naming convention between models, (2) special cases like removing some SBML elements during the merge, or (3) special cases like adding new SBML elements, manual intervention is required after the *AutoMerge* algorithm via the composition interface. In summary, *AutoMerge* can be thought of as a preliminary step, which solves simple merging issues while combining models; and, the modeler can then manually interfere and make changes to the composed model to ensure correctness. With the help of simulation tools, iModel and SimCom, simulation interface provides a sound, easy to use, pluggable, OS-independent, WYSIWYG web based solution for researchers to simulate computational models.

Our tool supports composition and simulation of models specified in SBML, up to SBML Level 3. Currently, the PathCase-SB simulation interface uses RoadRunner as its simulation engine. RoadRunner simulator is in active development (with issues and bugs being solved) and one of the most reliable simulators. Nonetheless, since PathCase-SB simulation interface is built on top of RoadRunner and with a well-specified and flexible connectivity, the system does have the ability to plug in another simulation engine (e.g., Jarnac, JSim, etc.) if and when it is needed in the future.

## Availability and requirements

•**Project name:** PathCase-SB Simulation and Composition Tools

•**Project home page:**http://nashua.case.edu/PathwaysSB/Web (site that hosts (BioModels models) and http://nashua.case.edu/PathwaysSBSBW/Web (site that hosts SBML Test Suite models)

•**Operating system(s):** Platform independent

•**Programming language:** ASP.NET Framework using the C#.NET language and Java for the visualization applet.

•**Other requirements:** JavaScript must be enabled in the browser. In order to view certain portions of the site correctly PathCase-SB need cookies enabled in the browser. In order to view the applet, version 1.6 (also known as version 6) or later of the Java Runtime Environment must be installed on the system from which the viewer is accessed. If the JRE is installed properly and the Graph Viewer still does not appear, the user should make sure that the browser’s security settings allow Java applets (or in the case of Internet Explorer, ActiveX controls). Best viewed at resolutions of 1024 × 768 pixels and up.

•**Any restrictions to use by non-academics:** Freely accessible.

## Competing interests

The authors declare that they have no competing interests.

## Authors’ contributions

Implementation and Tests: SAC, Wrote Manuscript: AEC, Revision Implementations: AEC, Supervised the Project: NL, RD, ZMO, GO. All authors read and approved the final manuscript.
